# Untangling the relationship between fat distribution, nutritional status and Parkinson’s disease severity

**DOI:** 10.1007/s40520-019-01166-x

**Published:** 2019-03-15

**Authors:** Maria S. Pisciotta, Domenico Fusco, Giulia Grande, Vincenzo Brandi, Maria R. Lo Monaco, Alice Laudisio, Graziano Onder, Anna R. Bentivoglio, Diego Ricciardi, Roberto Bernabei, Giuseppe Zuccalà, Davide L. Vetrano

**Affiliations:** 1 Department of Geriatrics, Fondazione Poliambulanza, Brescia, Italy; 2 Department of Geriatrics, Centro di Medicina dell’Invecchiamento, Università Cattolica del Sacro Cuore di Roma and IRCCS Fondazione Policlinico “A. Gemelli”, Rome, Italy; 3 Department of Neurobiology, Care Sciences and Society, Aging Research Center, Karolinska Institutet and Stockholm University, Tomtebodavägen 18A, Solna, Stockholm 17165 Sweden; 4 Department of Geriatrics, Campus Bio-Medico University, Rome, Italy; 5 Institute of Neurology, Università Cattolica del Sacro Cuore di Roma and IRCCS Fondazione Policlinico “A. Gemelli”, Rome, Italy; 6 “Don Gnocchi” Foundation, Milan, Italy

**Keywords:** Parkinson’s disease, Body composition, DXA, Nutritional status, Fat

## Abstract

**Background:**

Parkinson’s disease (PD) is responsible for significant changes in body composition.

**Aims:**

We aimed to test the association between PD severity and fat distribution patterns, and to investigate the potential modifier effect of nutritional status in this association.

**Methods:**

We enrolled 195 PD subjects consecutively admitted to a university geriatric day hospital. All participants underwent comprehensive clinical evaluation, including assessment of total and regional body composition (dual-energy X-ray absorptiometry, DXA), body mass index, nutritional status (Mini-Nutritional Assessment, MNA), motor disease severity (UPDRS III), comorbidities, and pharmacotherapy.

**Results:**

The fully adjusted linear regression model showed a negative association between UPDRS III and total body fat in kg and percentage (respectively,* B* − 0.79; 95% CI − 1.54 to − 0.05 and* B* − 0.55; 95% CI − 1.04 to − 0.05), percentage android fat (*B* − 1.07; 95% CI − 1.75 to − 0.39), trunk–leg fat ratio (*B* − 0.02; 95% CI − 0.04 to − 0.01), trunk–limb fat ratio (*B* − 0.01; 95% CI − 0.06 to − 0.01) and android–gynoid fat ratio (*B* − 0.01; 95% CI − 0.03 to − 0.01). After stratification by MNA score, all the parameters of android-like fat distribution resulted negatively associated (*p* < 0.001 for all) with UPDRS III, but only among subjects with a MNA < 23.5 (risk of malnutrition or malnutrition).

**Conclusion:**

We found a negative association between severity of motor impairment and total fat mass in PD, more specific with respect to an android pattern of fat distribution. This association seems to be driven by nutritional status, and is significant only among patients at risk of malnutrition or with overt malnutrition.

## Introduction

Aging and neurodegenerative diseases are both associated with qualitative and quantitative changes in body composition (i.e., muscle, bone and fat mass) [1, 2], and specific patterns of fat content and distribution have been described across neurodegenerative conditions [2]. People with Parkinson’s disease (PD), for example, have been reported to have higher prevalence of overweight and central obesity (i.e., visceral fat) in the early disease stages as compared with healthy controls [3]. Conversely, weight loss as well as low body mass index (BMI) dominate the latest stages of the disease in PD [4, 5], and have been associated with nigrostriatal depletion [6], impaired motor function [7], poor quality of life [8], and cognitive impairment [9].

Loss of body fat mass has been advocated as the main responsible factor for weight loss in PD [4], but little is known about the potential role of nutritional status as a potentially amendable mediator of such phenomenon. PD is indeed characterized by an altered balance between energy intake and expenditures, and an increased resting energy expenditure has been described, possibly related to rigidity and levodopa-induced dyskinesias [10]. Malnutrition is common during the progression of PD, affecting up to 24% of patients. Using screening tools (such as Mini-Nutritional Assessment, MNA) an even higher proportion of PD patients are identified as at risk of developing malnutrition [11]. An inadequate nutritional status may affect the relation between motor disturbances and weight loss in PD.

Furthermore, anorexia, gastrointestinal symptoms (e.g., nausea, constipation or delayed gastric emptying), depression, cognitive decline, loss of independence in activities of daily living (such as preparing meals) due to worsening motor performance, might all concur to reduced energy intake [12].

The aims of the present study were (1) to investigate the association between PD severity and several parameters of adiposity, paying special attention to the topology of fat distribution (android vs gynoid); and (2) to address the potential modifier effect of nutritional status in the association between PD severity and fat distribution.

## Methods

### Study participants

In this cross-sectional study, we analyzed 195 PD subjects consecutively admitted to the geriatric day hospital of the Catholic University of Rome, Italy, between January 1st, 2012 and December 31st, 2015. From an original sample of 213 subjects, fourteen have been excluded because of missing data on body composition and four because of missing data on nutritional status. PD was diagnosed in keeping with the United Kingdom Parkinson’s Disease Society Brain Bank criteria. Trained physicians evaluated all the participants. Data on demographics, functional status, diseases and drug treatment were properly collected. Fasting blood samples were obtained. All participants provided written informed consent, and the study protocol was previously approved from the Catholic University bioethics committee.

### Body composition evaluation

Body weight (Kg) and height (m) were measured in standard conditions in all participants, and body mass index (BMI) was properly computed and expressed in Kg/m^2^. All participants underwent body composition evaluation through dual-energy X-ray absorptiometry (DXA) on a daily automatic calibrated Hologic (Waltham, MA) system. Several parameters of total and regional adiposity were obtained through the automated embedded software algorithms [13]. We analyzed total fat (kg and % of body weight), android fat (kg and % of total body fat), and gynoid fat (kg and % of total body fat). The following parameters have been also considered: trunk–leg fat ratio, trunk–limb fat ratio and android–gynoid fat ratio as automatically provided by the system. Higher values of such ratios express a higher android-like fat distribution. Of notice, fat measured from a DXA whole-body scan is highly correlated and linearly related to visceral fat measurements carried out through computed tomography [14].

### Nutritional status evaluation

Participants’ nutritional status was evaluated through the Mini-Nutritional Assessment (MNA) [15]. This tool includes 18 items evaluating anthropometric, functional, clinical and dietary parameters, composing a total score as high as 30. According to MNA, scoring of < 23.5 suggests a malnutrition risk, and a score of < 17 overt malnutrition. MNA is a validated screening tool suited for the evaluation of the nutritional status in older adults. It has been shown to provide reliable evaluations across different populations and care settings and to be useful in predicting several outcomes [16]. Finally, MNA has been indicated as a reliable tool for the evaluation of malnutrition and risk of malnutrition in people with PD [17–19].

### Covariates

Data on age, gender and education were collected through a standard questionnaire. Time from diagnosis of PD was expressed in years. The severity of PD was assessed using the Unified Parkinson’s Disease Rating Scale (UPDRS) part III that evaluates the cardinal symptoms of the disease, thus staging the level of motor impairment. All the evaluations were carried out during the participant’s “on” phase, within 2 h from the last antiparkinsonian drug administration. The L-dopa equivalent daily dose (LEDD) was finally obtained and indexed by body weight, transforming the antiparkinsonian drug daily doses in L-dopa equivalents through a previously described algorithm [20]. Cognition was assessed using the Mini-Mental State Examination (MMSE), scoring 0–30, with higher scores indicating better performance. Functional ability was estimated using the Katz’s activities of daily living (ADLs), scoring 0–6, with lower scores indicating higher dependency. Depressive symptoms were assessed using the validated Italian version of the 15-item Geriatric Depression Scale (GDS). Fasting blood samples were obtained from all participants. In the present study, erythrocyte sedimentation rate (ESR; mm/s) was analyzed and used as an inflammatory parameter. Drugs were coded according to the Anatomical Therapeutic and Chemical codes. Diagnoses were coded according to the International Classification of Diseases, ninth edition, Clinical Modification codes.

### Statistical analysis

Variables are presented as mean values ± standard deviation (SD) or median and Inter Quartile Range (IQR) and absolute numbers and percentages (%), and properly compared between participants with an UPDRS III below and above the median value (24, IQR 18–31). Analysis of variance (ANOVA) and Chi-square test were used to compare continuous and categorical variables normally distributed, respectively, and non-parametric tests were used for non-normally distributed variables. The crude correlation (*R*^2^ coefficient of determination) between all the adiposity parameters and UPDRS III was reported in scatter-plot figures according to a MNA cut-off value of 23.5. The association (*B* and 95% confidence interval [95% CI]) between all the adiposity parameters and UPDRS III (every five points) was tested through three different linear regression models adjusted for potential confounders. The additive statistical interaction between MNA and UPDRS III was tested and analyses subsequently stratified according to MNA values above and below the score of 23.5. A *p* value of < 0.05 was considered significant. Analyses were carried out through SPSS for Windows 18.0 (SPSS Inc., Chicago, IL).

## Results

**Fig. 1:**
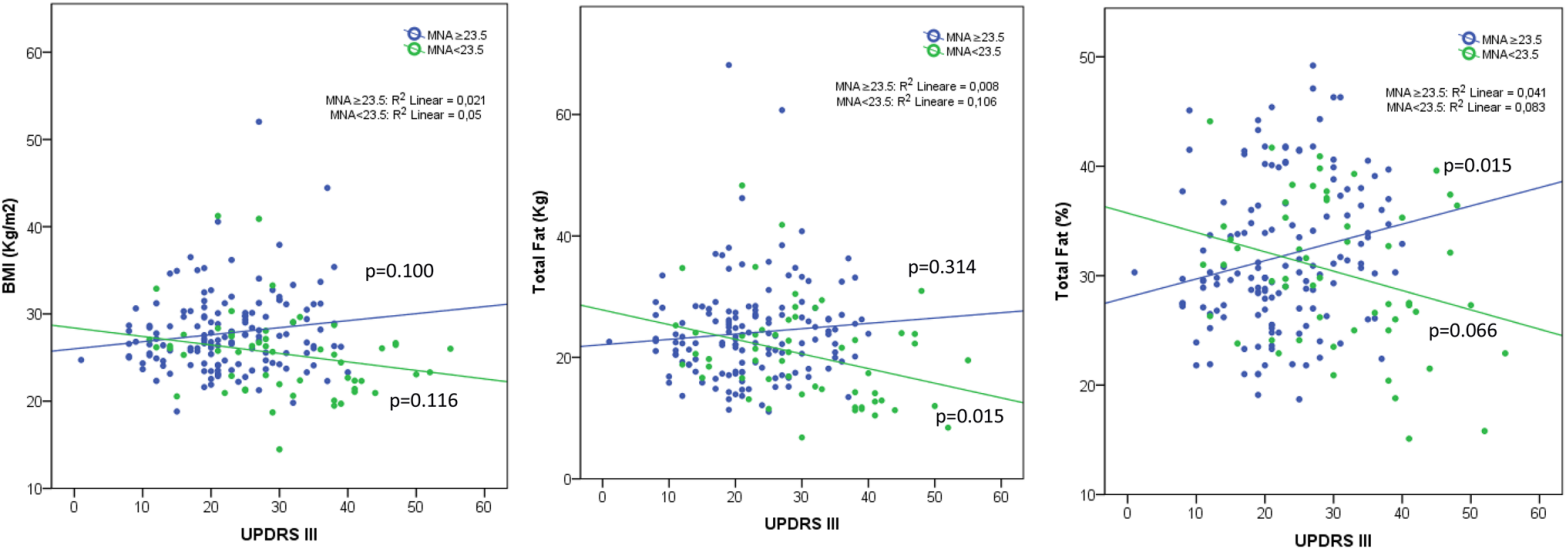
Correlation between total body fat measures and UPDRS III by Mini-Nutritional Assessment score

**Fig. 2:**
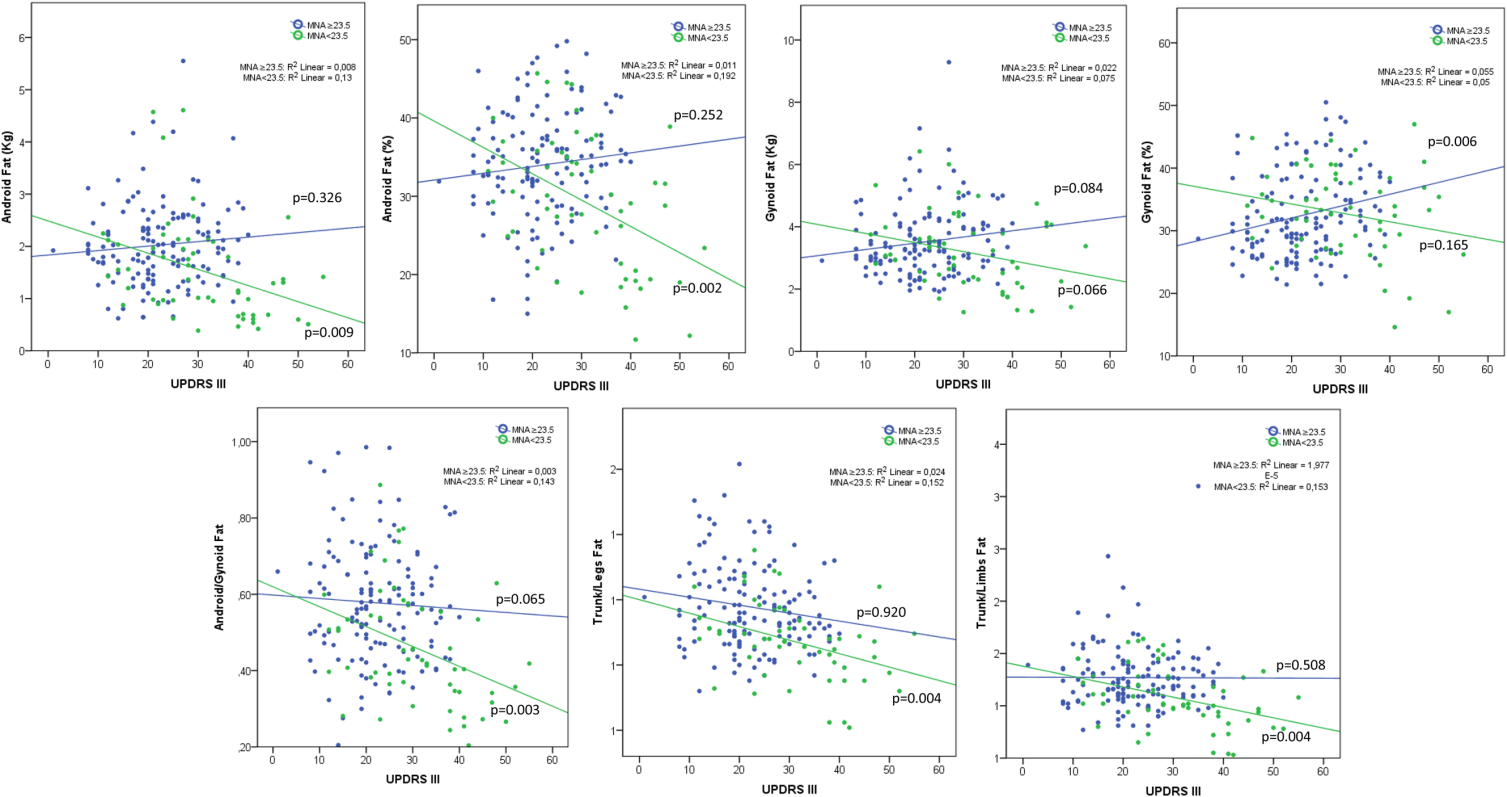
Correlation between district fat distribution and UPDRS III by Mini-Nutritional Assessment score

Among the study participants the mean age was 73.6 ± 7.2 years, 71 (36%) were females and 56 (29%) presented with a MNA score of < 23.5. As shown in Table 1, compared with subjects with a UPDRS above the median, those with a UPDRS below the median were more likely male, more educated and presented with better cognitive function, mood, functional status, and nutritional status. Also, they presented with lower ESR values and comorbidities. As shown in Table 2, subjects with an UPDRS III below the median presented with higher trunk-–leg and trunk–limb ratios. As shown in Table 3, according to the fully adjusted linear regression model, UPDRS III was negatively associated with total body fat in kg and as % (respectively,* B* − 0.79; 95% CI − 1.54 to − 0.05 and* B* − 0.55; 95% CI − 1.04 to − 0.05), android fat as % (*B* − 1.07; 95% CI − 1.75 to − 0.39), trunk–leg fat ratio (*B* − 0.02; 95% CI − 0.04 to − 0.01), trunk–limb fat ratio (*B* − 0.01; 95% CI − 0.06 to − 0.01), and android–gynoid fat ratio (*B* − 0.01; 95% CI − 0.03 to − 0.01). After entering the MNA score in the adjusted model, only android fat as % and trunk–leg fat ratio were still negatively associated with UPDRS III, but the association was weaker. Figures 1 and 2 show the correlation between all the adiposity parameters and UPDRS III stratified by the MNA. According to such analyses, all the parameters indicating an android-like fat distribution resulted negatively associated (*p* < 0.001 for all) with UPDRS III, but only among subjects with a MNA < 23.5. The interaction analysis showed a statistical additive interaction between the UPDRS III and the MNA score for most of the adiposity parameters (see Table 4). In the stratified analyses, all the adiposity parameters resulted negatively associated with UPDRS III only among participants with a MNA score < 23.5. Conversely, gynoid fat as % was positively associated with UPDRS III among subjects with a MNA ≥ 23.5.

**Table 1:** Main characteristics of participants according to the median UPDRS III score (below or above the median value)

	UPDRS III < 24 *N* = 97 (50%)	UPDRS III ≥ 24 *N* = 98 (50%)	*p*
Age, mean ± SD	73.1 ± 6.7	74.2 ± 7.7	0.261
Sex (female), *n* (%)	26 (27)	45 (46)	0.006
Education (years), mean ± SD	11.9 ± 4.7	9.6 ± 5.1	0.001
Mini-Mental State Examination, mean ± SD	27.2 ± 2.2	25.6 ± 3.9	< 0.001
Geriatric Depression Scale, mean ± SD	4.6 ± 3.0	6.0 ± 3.7	0.004
Activities of daily living, mean ± SD	5.1 ± 1.0	4.0 ± 1.6	< 0.001
Mini-Nutritional Assessment, mean ± SD	25.4 ± 3.0	23.4 ± 3.6	< 0.001
Erythrocyte sedimentation rate (mm/s), median (IQR)	9 (7–16)	13 (7–25)	0.030
Years from Parkinson’s diagnosis, median (IQR)	2.9 (0.8–6.8)	4 (1.9–7.8)	0.400
Levodopa equivalent daily dose (mg/kg), mean ± SD	8.0 ± 5.7	8.2 ± 5.1	0.741
Number of chronic comorbidities, mean ± SD	3.0 ± 1.8	3.7 ± 2.0	0.007
Number of drugs, mean ± SD	5.8 ± 2.8	6.2 ± 2.9	0.353

**Table 2:** Association (*B* and 95% confidence intervals) between the UPDRS III (every five-point increase) and adiposity parameters

	UPDRS III < 24 *N* = 97 (50%)	UPDRS III ≥ 24 *N* = 98 (50%)	*P*
Body mass index (kg/m^2^), mean ± SD	27.4 ± 4.0	26.9 ± 5.4	0.503
Total fat (kg), mean ± SD	23.6 ± 8.2	22.4 ± 8.5	0.318
Total fat (%), mean ± SD	30.8 ± 6.3	32.0 ± 7.4	0.260
Android fat (kg), mean ± SD	1.9 ± 0.8	1.8 ± 0.9	0.222
Android fat (%^a^), mean ± SD	33.4 ± 7.0	32.2 ± 8.4	0.297
Gynoid fat (kg), mean ± SD	3.4 ± 1.0	3.4 ± 1.3	0.962
Gynoid fat (%^¥^), mean ± SD	31.8 ± 6.1	33.5 ± 7.5	0.081
Trunk–leg ratio, mean ± SD	1.0 ± 0.1	0.9 ± 0.2	0.001
Trunk–limb ratio, mean ± SD	1.3 ± 0.3	1.2 ± 0.3	0.016
Android–gynoid fat ratio, mean ± SD	0.6 ± 0.2	0.5 ± 0.2	0.503

^a^Percentage of the total region mass

**Table 3:** Association (*B* and 95% confidence intervals) between the UPDRS III (every five-point increase) and adiposity parameters

	Age, sex and education adj.			Fully adj.^a^			Fully adj + MNA		
	B	95% CI		B	95% CI		B	95% CI	
		Lower limit	Upper limit		Lower limit	Upper limit		Lower limit	Upper limit
Body mass index	− 0.21	− 0.56	0.15	− 0.22	− 0.65	0.21	− 0.01	− 0.43	0.42
Total fat (kg)	− 0.70	− 1.31	− 0.09	− 0.79	− 1.54	− 0.05	− 0.51	− 1.26	0.24
Total fat (%)	− 0.46	− 0.87	− 0.05	− 0.55	− 1.04	− 0.05	− 0.35	− 0.84	0.15
Android fat (kg)	− 0.73	− 0.14	− 0.10	− 0.08	− 0.15	0.01	− 0.04	− 0.12	0.03
Android fat (%^b^)	− 1.02	− 1.58	− 0.47	− 1.07	− 1.75	− 0.39	− 0.74	− 1.41	− 0.07
Gynoid fat (kg)	− 0.06	− 0.14	0.02	− 0.09	− 0.19	0.01	− 0.05	− 0.15	0.05
Gynoid fat (%^b^)	− 0.22	− 0.59	0.16	− 0.35	− 0.80	0.10	− 0.23	− 0.69	0.23
Trunk–leg fat	− 0.02	− 0.04	− 0.01	− 0.02	− 0.04	− 0.01	− 0.02	− 0.03	− 0.01
Trunk–limb fat	− 0.03	− 0.06	− 0.01	− 0.03	− 0.06	− 0.01	− 0.02	− 0.05	0.01
Android–gynoid fat	− 0.02	− 0.03	− 0.01	− 0.01	− 0.03	− 0.01	− 0.01	− 0.02	0.01

^a^Adjusted for age, sex, education, Mini-Mental State Examination, and Geriatric Depression Scale, activities of daily living, erythrocyte sedimentation rate, and number of comorbidities

^b^Percentage of total fat

**Table 4:** Association (*B* and 95% confidence intervals) between UPDRS III (every five-point increase) and adiposity parameters stratified by MNA

	MNA ≤ 23.5 *N* = 56 (29%)			MNA > 23.5 *N* = 139 (71%)			Interaction^a^
	B	95% CI		B	95% CI		
		Lower limit	Upper limit		Lower limit	Upper limit	
Body mass index	− 0.87	− 1.61	− 0.12	0.34	− 0.19	0.87	*0.009*
Total fat (kg)	− 2.11	− 3.33	− 0.89	0.33	− 0.62	1.28	*0.012*
Total fat (%)	− 1.70	− 2.65	− 0.76	0.37	− 0.19	0.92	< *0.001*
Android fat (kg)	− 0.21	− 0.35	− 0.07	0.04	− 0.05	0.14	*0.010*
Android fat (%^¥^)	− 2.51	− 3.77	− 1.25	0.10	− 0.66	0.86	*0.001*
Gynoid fat (kg)	− 0.29	− 0.47	− 0.10	0.09	− 0.03	0.02	*0.002*
Gynoid fat (%^b^)	− 1.65	− 2.57	− 0.72	0.58	0.10	1.07	< *0.001*
Trunk–leg fat	− 0.03	− 0.05	− 0.01	− 0.02	− 0.04	0.01	*0.941*
Trunk–limb fat	− 0.06	− 0.10	− 0.02	− 0.01	− 0.04	0.03	*0.372*
Android–gynoid fat	− 0.02	− 0.04	− 0.01	− 0.01	− 0.02	0.01	*0.297*

Model adjusted for age, sex, education, Mini-Mental State Examination, Geriatric Depression Scale, activities of daily living, erythrocyte sedimentation rate, and number of comorbidities

^a^Additive interaction (*p* value) between the Mini-Nutritional Assessment and UPDRS III

^b^Percentage of total fat

## Discussion

In this sample of older adults with PD we observed a negative association between severity of motor impairment and fat mass, selectively with android-like distributed fat. The association remained significant after adjusting for several confounders, but not after adjusting for nutritional status. After stratification, the association between motor severity of PD and measures of adiposity was confirmed only among people at risk of malnutrition, or with overt malnutrition.

To the best of our knowledge, this is the largest sample of people with PD which body composition has been assessed through DXA scan. Weight loss is common in neurodegenerative diseases, and can be due to loss of energy balance. This may be related either to primary neuronal dysfunction and neurodegeneration (such as olfactory and taste loss, impairment of hypothalamic regulation of appetite and thermoregulation, cognitive decline, depression) or to secondary factors, such as disability and drugs’ side effects [17, 21]. Weight loss in PD may present soon after the diagnosis; it has been related to loss of body fat [22], even if concomitant reduced lean mass cannot be excluded [23]. However, data on fat distribution through the course of disease are discordant. In the early stage of PD, an increase in body weight (mainly as visceral fat) has been reported [3]. Indeed, Bernhardt et al. recently demonstrated a higher visceral to sub-cutaneous fat ratio in parkinsonian subjects when compared to healthy controls, using MRI [24].

Generalized sympathetic denervation is common in PD [25–27], even in the early phase of disease. Autonomic dysfunction could be responsible for greater fat deposition since sympathetic denervation leads to chronotropic insufficiency and reduced thermogenesis, thus reducing energy expenditure [28]. Therefore, after an initial increase in body weight, a progressive weight loss occurs [4], more pronounced in the advanced stages of disease [19]. In our sample, worse motor performances (as assessed by UPDRS) were associated with reduced fat mass. This association was stronger with android fat. This is in line with previous observations [7, 11].

The main result of our study is that nutritional status drives the association between total and regional adiposity and disease severity in Parkinson’s disease patients. Recent studies have suggested a potential neuroprotective role of adipocytokines. Reduced levels of leptin, a cytokine released by adipocytes, were observed by some authors in PD patients experiencing weight loss. Reduced levels of leptin reported in PD people with weight loss might explain the relationship between worse motor function and low fat mass [29, 30]. Beyond its contribution to body weight, the qualitative distribution of fat mass has several implications worthy to be mentioned. Android fat distribution has been related to increased cardiovascular disease risk, mediated by hyperlipidemia and hyperglycemia [31]. Indeed, insulin resistance has been associated with a slight increase in risk of PD, through a suppressive action on dopaminergic neurons in substantia nigra [32]. The reduced CV risk described in people with PD, and associated with both disease severity and duration, may be partially mediated by the reduction in android adiposity we describe in the present paper, at least for subjects at risk of malnutrition.

Like weight loss, malnutrition is common in PD [11] and many possible causes can contribute to its occurrence: hyposmia, reduced appetite, altered reward mechanism due to degeneration in meso-corticolimbic network, reduced levels of orexin, could all account for undernutrition, but none of them has been consistently related to weight loss [33]. Increased energy expenditure has been observed in PD patients with worsening motor performance [34], and its main determinants are considered dyskinesias and rigidity. Along with gastrointestinal symptoms (such as sialorrhea, dysphagia and constipation), leading to reduced energy intake and malnutrition [12], disability, cognitive impairment, and depression are also significant risk factors for malnutrition in the elderly population and should be taken into consideration [35].

In our study, after adjusting for possible confounders, the association of UPDRS and fat mass remained statistically significant only for the subgroup of patients with lower MNA scores. In this group, higher fat mass (particularly android fat) could represent an index of better nutritional status. In other words, a good nutritional status might protect PD patients from weight loss associated with disease severity. Maintaining a good nutritional status might potentially slow both weight loss and motor impairment. Screening tools, such as MNA, may allow early detection of subjects with PD at risk for malnutrition and proper personalized nutritional intervention [16, 17]. Individualized dietary counseling should be offered to patients, taking into consideration the specific clinical context, disease duration, level of motor impairment and comorbidities.

To note, a cross-talk between fat and muscle has been demonstrated by several studies. A number of signaling proteins, produced by muscle and fat cells take part to this talk and are responsible for the changes in body composition observed with age [36]. Interestingly, in older people, along with body weight changes, a common consequence of malnutrition is represented by sarcopenia, the progressive decline in muscle mass and muscle function. In a recent study carried out on the same study population of the present report, we showed that in PD patients, sarcopenia is associated with more than twofold higher odds of poor motor function, as measured through the UPDRS [37]. The potential synergism between muscle and fat changes and their impact on motor function in PD requires to be addressed in future longitudinal studies.

Some limitations of the present study need to be acknowledged. First, as previously stated, the cross-sectional nature does not allow firm conclusions either on causality or timing among the observed phenomena. Second, we analyzed a non-random sample of elderly people with PD, admitted to a single care center; this may limit the generalizability of our results. Third, functional or biological parameters not collected in the present study might account for a residual confounding, potentially affecting our results.

In conclusion, our results suggest that nutritional status plays a relevant role in driving the relationship between body composition (particularly fat content and distribution) and motor function in people with PD. The detrimental effect of weight loss on motor function might be prevented by maintaining a proper nutritional status, avoiding malnutrition. In this regard, the early detection of malnutrition or risk of malnutrition in subjects with PD is warranted. Such findings deserve further investigation through longitudinal studies.
